# Difficulties of Using Single-Diseased Guidelines to Treat Patients with Multiple Diseases

**DOI:** 10.3389/fpubh.2015.00067

**Published:** 2015-04-29

**Authors:** Sarah Dörenkamp, Ilse Mesters, Joep Teijink, Rob de Bie

**Affiliations:** ^1^Department of Epidemiology, CAPHRI School for Public Health and Primary Care, Maastricht University, Maastricht, Netherlands; ^2^Center for Evidence-Based Physiotherapy (CEBP), Maastricht University, Maastricht, Netherlands; ^3^Department of Vascular Surgery, Catharina Hospital, Eindhoven, Netherlands

**Keywords:** clinical practice guidelines, evidence-based practice, comorbidity, physical therapy modalities, clinical decision making

## Difficulties of Using Single-Diseased Guidelines to Treat Patients with Multiple Diseases

Discipline- and disease-specific medical care is increasingly underpinned by evidence-based guidelines. The main goal of these guidelines is to give the best available diagnostic and treatment advice, to decrease variability in daily clinical practice, to reduce inappropriate practice, and to improve cost-effectiveness, ideally resulting in better health outcomes for patients (1). For example, the Royal Dutch Society for Physical Therapy (KNGF) and the Dutch College of General Practitioners (NHG) have published 17 and 96 disease-specific guidelines, respectively, all with the aim of providing a stronger scientific foundation and improve health care (2, 3). However, the majority of patients [40% at the age of 50 years and at least two-thirds of the octogenarian population (4)] simultaneously suffer from multiple medical problems. Caregivers, therefore, need to consider single-disease evidence contained in a guideline in the view of other relevant guidelines. In this matter, Hurwitz et al. (5) already warned that guidelines may encourage users to apply recommendations “*rigidly or unthinkingly*,” even in situations where departure from a guideline would be desirable, as might be the case with multimorbidity. Thus, when treating multi-diseased patients, physical therapists (PT) themselves have to design a customized treatment plan based on guidelines developed for patients with only one disease and in consideration of the impact of multimorbidity. Once a patient with multimorbidity is admitted to a physical therapy practice, the therapist may consider multiple treatment paths. This situation could be compared with a maze: a complex branching passage with many different pathways. To solve the maze, the PT must find a route to travel from start to finish.

## The Maze of Multimorbidity

The maze of multimorbidity could be entered at different points based on the disease (e.g., type, severity) and the health needs for which the patient has been referred to the PT.

**Figure 1:**
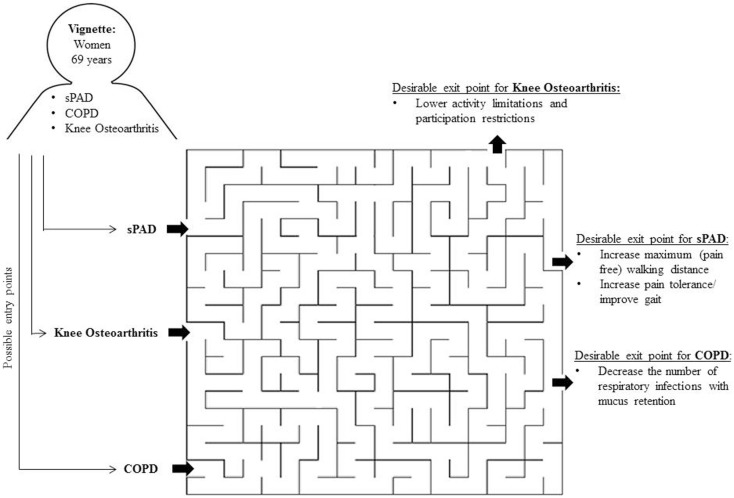
**The maze of multimorbidity**.

Imagine that a patient with intermittent claudication [IC: a symptom of symptomatic Peripheral Arterial Disease (sPAD); see vignette in Figure 1] was not referred to the PT for the treatment of sPAD, but by a pulmonologist for the treatment of chronic obstructive pulmonary disease (COPD). Consequently, the treatment focus would not be aimed at increasing the maximum walking distance, but instead at reducing dyspnea and improving mucus clearance.

The maze of multimorbidity also has different final and intermediate *exit* points (i.e., desired treatment outcomes). The final outcome is set by the disease for which the patient was referred. For example, if a patient with sPAD visits the PT with the aim to increase the maximum walking distance, the desirable outcome of physical therapy treatment could be an increase in meters that the patient can walk. Imagine that this patient would be hindered while walking by shortness of breath due to COPD. Then, the intermediate goal for physical therapy care would be an improvement in breathing techniques. After the breathing problems have been reduced, the PT may continue treating the index disease sPAD. If the PT continues treating the patient without taking heed of the observed shortness of breath, the patient will never reach the desired treatment outcome. The patient will always be hindered at the same point because he or she is walking into a dead end due to limitations related to COPD.

## Experience-Based Evidence: A Solution in Cases of Multimorbidity?

The maze metaphor illustrates that clinical decisions in physical therapy require considering a discrete range of choices from which the PT will select the most appropriate treatment or intervention to meet a patient’s specific needs. Ideally, we make these choices based on the highest value of level 1a evidence from research. However, complex patients suffering from multimorbidity are under-represented in systematic reviews and randomized controlled trials (RCTs) because their presence presents methodological challenges (6). Hence, collecting evidence on all possible disease combinations might even be an impossible endeavor. The high level of heterogeneity in the study population would require researchers to recruit impossibly large study populations. Taking into account loss to follow-up and drop-out rates, required sample sizes will enlarge.

How to proceed from here? Relying on experience-based evidence might be an option, to underpin current practice. For example, Moitra et al. (8) elaborated on the impossibility to perform large epidemiological studies in case of perioperative cardiac arrest, a disease that occurs rarely, has a heterogeneous spectrum of causes and is distinct from cardiac arrest in other settings. The authors highlighted that from a practical point of view, it is not feasible to avoid anesthesia during surgical manipulation in order to be able to investigate the contribution of anesthesia to cardiac arrest. However, among anesthesiologists, there is much expertise and experience in managing cardiac arrest in perioperative patients and thus experience-based evidence should be cherished in the generation of protocols. But, relying on care providers, experience has also raised discussion in many different fields in medicine (8–10). Rinchuse et al. (7), for instance, from the field of Orthodontic and Dentofacial Orthopedics, warn for incautiously trusting personal perceptions (empiricism), highly respected people (authority), intuitively appealing logical ideas (rationalism), and long held beliefs (tenacity). Although these pitfalls exist, Leape et al. (9) from the field of Health Policy and Management stated that there will never be complete evidence for everything, but that the alternative is to make reasonable judgments based on best available evidence in combination with successful experiences in health care.

Acknowledging the pitfalls of experience-based information, it may be helpful when large numbers of data are collected and analyzed in a systematic way. In general, identifying the specific influence of comorbidities on treatment outcome, in addition to considering PTs’ experiences working with these patients in the past, may enable us to identify possible alternations of treatment for these patient groups and therefore improve treatment success. Health professionals gradually acquire experiences that enable them to make implicit connections between patients who need to be treated and patients they have seen in the past. In the context of systematically gathered experience-based evidence, curve matching may be a powerful approach that can enhance the memory of health professionals by putting existing information (i.e., electronic medical record data collections) to a new use. The key idea is to find relevant historic data about patients who are similar to the new patient and to use this data to suggest the treatment outcome of the new patient (6, 11). This approach takes into account the uniqueness of patients by including individual patient characteristics and specific disease combinations in the treatment outcome determination. It gives PTs the opportunity to customize medical treatments for individual patients. Hence, putting existing information to a new and systematic use may help guide PT through the complex process of managing patients with multimorbidity.

## Author Contributions

All authors contributed to his work. The ideas of the manuscript were discussed with the whole project team. SD, IM, and RB wrote the manuscript. JT commented on the manuscript and gave conceptual advice at the final stage.

## Conflict of Interest Statement

We declare that the research was conducted in the absence of any commercial or financial relationships that could be construed as a potential conflict of interest. This manuscript has not been previously published and is not under consideration in the same or substantially similar form in any other peer-reviewed media. We declare that we have no significant competing financial, professional, or personal interests that might have influenced the performance or presentation of the work described in this manuscript. All authors listed have contributed sufficiently to the project to be included as authors.

## Abbreviations

COPD, chronic obstructive pulmonary disease; IC, intermittent claudication; PT, physical therapist; Spad, symptomatic peripheral arterial disease.
